# Molecular Mechanisms of Host Cytoskeletal Rearrangements by *Shigella* Invasins

**DOI:** 10.3390/ijms151018253

**Published:** 2014-10-10

**Authors:** Jun Hyuck Lee, HaJeung Park, Yong Ho Park

**Affiliations:** 1 Division of Polar Life Sciences, Korea Polar Research Institute, Incheon 406-840, Korea; 2 Department of Polar Sciences, University of Science and Technology, Incheon 406-840, Korea; 3 The Scripps Research Institute, Scripps Florida, 130 Scripps Way, Jupiter, FL 33458, USA; 4 Laboratory of Veterinary Microbiology, College of Veterinary Medicine, Seoul National University, Seoul 151-742, Korea; E-Mail: yhp@snu.ac.kr

**Keywords:** actin, bacillary dysentery, bacterial proteins, invasin, review, *Shigella*

## Abstract

Pathogen-induced reorganization of the host cell cytoskeleton is a common strategy utilized in host cell invasion by many facultative intracellular bacteria, such as *Shigella*, *Listeria*, enteroinvasive *E. coli* and *Salmonella*. *Shigella* is an enteroinvasive intracellular pathogen that preferentially infects human epithelial cells and causes bacillary dysentery. Invasion of *Shigella* into intestinal epithelial cells requires extensive remodeling of the actin cytoskeleton with the aid of pathogenic effector proteins injected into the host cell by the activity of the type III secretion system. These so-called *Shigella* invasins, including IpaA, IpaC, IpgB1, IpgB2 and IpgD, modulate the actin-regulatory system in a concerted manner to guarantee efficient entry of the bacteria into host cells.

## 1. Introduction

The actin cytoskeleton plays a major role in nearly every aspect of biological processes, such as cell division, cell morphogenesis and motility, neurite growth, immune synapse formation, endocytosis, phagocytosis and intracellular protein trafficking [1,2,3,4,5,6,7]. Actin dynamics are regulated through conserved signaling processes, most notably involving the Rho family GTPases Rho, Rac and Cdc42 [8,9]. The signals transmitted through these “hubs” control actin-binding proteins and scaffold proteins to modulate actin polymerization, bundling and depolymerization. The actin cytoskeleton and its regulatory system are often exploited by microbial pathogens to attach onto and invade host cells [10,11,12,13,14,15,16]. Certain Gram-negative pathogens do so by delivering pathogenic effector proteins into the host cell through the type III secretion system (T3SS) (Table 1).

**Table 1:** Type III secretion system (T3SS) effectors that induce actin rearrangement during infection.

Gram (−) Bacteria	Effectors
*Shigella* spp*.*	IpaA [17,18], IpaC [19,20], IpgB1/IpgB2 [21,22] and IpgD [23,24]
*Salmonella* spp.	SipA [25,26], SipC [13], SopE [27,28], SopB [29,30,31] and SpvB [32,33]

*Shigella*, the etiologic agent of bacillary dysentery, is an enteroinvasive facultative intracellular pathogen that is highly adapted to human colonic epithelial cells [34,35]. Bacillary dysentery is manifested by bloody mucous stool resulting from damage to the epithelial cells of the lower intestine, especially in the region of the sigmoid colon [36]. *Shigella* infection is self-limiting in otherwise healthy adults, but can be deadly in young children and elderly people. *Shigella* lacks both flagella and adherence factors. Thus, breach of the epithelial cell layer occurs through M-cells and macrophages in Peyer’s patches [37]. On entry into the submucosa, *Shigella* induces macropinocytosis to invade the basolateral region of the epithelial cells [37]. It escapes from the endocytic vacuole, multiplies and transfects intercellularly to damage the epithelial layer [38]. In the early stage of infection, the intimate bacterial contact site of the epithelial cell is enriched with actin nucleation. Subsequently, the actin pool is depolymerized at the contact region, while the area surrounding the region generates filopodia through heavy actin polymerization [39]. The extensive rearrangement of the actin cytoskeleton during this step is directed by a subset of T3SS effectors, that is IpaA, IpaC, IpgB1, IpgB2 and IpgD [17,19,21,22,40,41]. These invasins act on multiple targets of the actin regulatory system to guarantee comprehensive and efficient entry of the bacteria. IpaA binds to and activates the focal adhesion protein, vinculin, by severing the intramolecular head-tail interaction through the bundle conversion mechanism [18]. Activation of vinculin in this manner triggers actin depolymerization through modulation of its partial capping activity [42]. Together with IpaB, IpaC constructs the translocation channel for effectors on the surface of the host cell membrane [43]. IpaC is also involved in massive actin polymerization during *Shigella* invasion [19]. IpgB1 and IpgB2 belong to a family of virulence effectors that share the WxxxE sequence motif and are known to have guanine-nucleotide exchange (GEF) activity for Rho GTPases [21]. The GEF activity of IpgB1 and IpgB2 is essential for modulating the host cell cytoskeleton and membrane ruffle formation during bacterial invasion [21,44]. IpgD is a potent inositol 4-phosphatase that causes accumulation of phosphatidylinositol 5-phosphate (PI(5)P), which is thought to be responsible for the decrease in membrane tether force, the formation of membrane blebbing and the remodeling of actin filaments [23]. This review summarizes the recent advances pertaining to *Shigella* invasins, focusing on the mechanisms of the invasins that modulate the actin cytoskeleton and its polymerization system.

## 2. IpaA: Mimicry of the Focal Adhesion Protein, Talin

**Figure 1:**
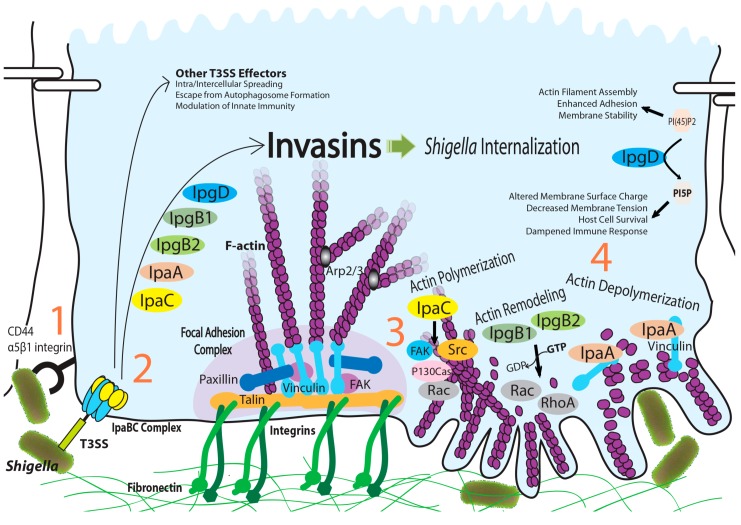
Overview of *Shigella* internalization with the action of T3SS invasins injected into the host cell cytosol. Targeting of key regulators in actin dynamics by invasins is highlighted in this schematic. Internalization of *Shigella* is accomplished by (1) attachment to host cells by an interaction between the IpaB/C complex and CD44/α5β1 integrins; (2) injection of T3SS invasins; (3) disassembly of focal adhesion complexes, microspike formation and actin remodeling and (4) actin depolymerization and ruffle formation. The sequence of events occurs at the intimate bacterial contact site.

Focal adhesion complexes anchor epithelial cells to the extracellular matrix [45]. The stability of a focal adhesion complex in the cytosol is tightly controlled by integrins, adaptor proteins and signaling proteins. Talin is a major focal adhesion adaptor protein involved in the formation and stability of focal adhesions by linking integrins to the actin filaments of the cytoskeleton [46] (Figure 1).

**Figure 2:**
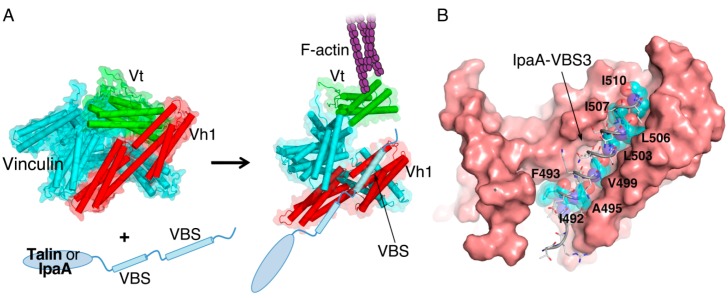
Vinculin activation by IpaA or talin. (**A**) Inactive vinculin is in a closed conformation through the Vh1:Vt interaction (**left**). IpaA or talin binds to Vh1 of vinculin through their vinculin-binding sites (VBSs), inducing helical bundle conversion of Vh1 (**right**). The structural change causes dissociation of the Vh1:Vt interaction, which triggers F-actin binding to Vt. Activated vinculin (**right**) is modeled from an inactive vinculin structure (**left**) (PDB entry: 1TR2). The schematic model shows only two VBSs, although talin has 11 VBSs [54]; (**B**) The crystal structure of IpaA-VBS3/vinculin complex (PDB entry: 3RF3) is shown as a representative example if the VBS/vinculin interaction. The hydrophobic residues of VBS3 that are involved in the interaction with vinculin are shown as cyan van der Waals spheres with residue labels. Vinculin is shown as a surface model.

Vinculin is another adaptor protein involved in the stability of focal adhesions. The domain organization of vinculin includes globular head domains (Vh1 to Vh5) followed by a tail domain (Vt) linked through a proline-rich region. Vh1 of vinculin contains binding sites for talin and α-actinin, while Vt contains F-actin- and raver1-binding sites [47,48,49,50]. Recent crystal structures of full-length vinculin show that these binding sites are masked by an intramolecular Vh1-Vt interaction [51,52] (Figure 2). Vinculin binding sites (VBSs) are amphipathic helices that are 19 amino acids in length and are found in vinculin-binding proteins, such as talin and α-actinin [47,48]. Crystal structures of Vh1 with various talin-VBS complexes have shown that a VBS insertion in the four-helical bundle of Vh1 induces a conformational change, thereby inhibiting the Vh1-Vt interaction [53]. Activation of vinculin is critically dependent on the VBS to displace Vt from Vh1, thereby exposing the binding sites for focal adhesion components (Figure 2).

**Figure 3:**
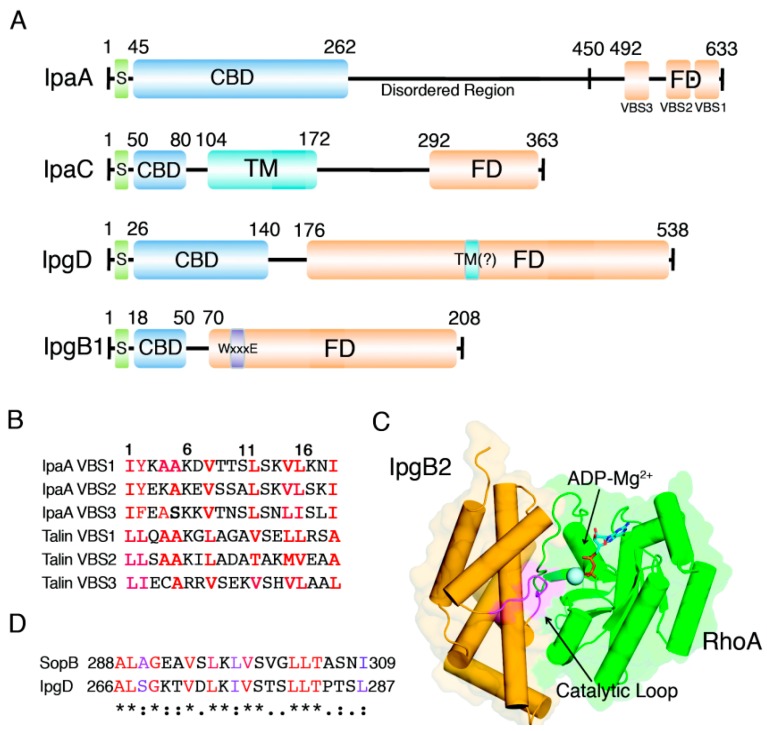
Primary and tertiary structures of *Shigella* invasins. (**A**) Schematic diagrams of the domain organizations of *Shigella* invasins for which tertiary structural information is not yet available. S, signal sequence; CBD, chaperone-binding domain; FD, functional domain; TM, transmembrane domain; (**B**) Sequence alignment of selected VBSs. Conserved amino acid residues important for the recognition of Vh1 are red. The numbers at the top of the alignments represent the amino acid positions of VBSs; (**C**) Crystal structure of IpgB2 in complex with RhoA (PDB entry: 3LW8). The catalytic loop is in magenta; (**D**) Sequence alignment of putative TM domains of SopB and IpgD.

It is not surprising to find a pathogenic effector that attaches to the focal adhesion complex, as *Shigella* invasion is initiated at the basolateral region of epithelial cells, where cell-matrix interactions are formed. IpaA is a *Shigella* invasin that is involved in this process by interfering with the vinculin:talin interaction at focal adhesion sites [18]. An IpaA mutant strain has been found to have an ~10-fold decrease in its ability to invade HeLa cells, and the activity of IpaA has been reported to depend on its vinculin-binding capability [17]. The primary structure of IpaA contains an amino-terminal chaperone-binding domain, a highly disordered middle region and an all-helical carboxy-terminal region. The last two carboxy-terminal helices in IpaA are tandem VBSs through which IpaA binds to the Vh1 domain of vinculin for its activation [18] (Figure 3A). Recently, we reported another VBS in IpaA. The discovery of a new VBS in IpaA highlights the resemblance of IpaA to talin [55]. The sequences of IpaA VBSs are highly identical to those in talin (Figure 3B). In addition, the multiple VBSs in IpaA are reminiscent of talin. While most of the talin VBSs are cryptic and have low affinity for vinculin, IpaA VBSs are constitutively active. Furthermore, the tandem arrangement of IpaA VBSs markedly increases the affinity for vinculin [18]. IpaA appears to activate vinculin more efficiently than talin does. However, competition assay analysis through native-PAGE by Izard *et al.* showed that IpaA does not efficiently displace a pre-existing vinculin:talin complex [18]. The authors predicted that IpaA reduces inactive pools of vinculin. On the other hand, observation of the dynamic changes in focal adhesion after microinjection of IpaA into HeLa cells clearly demonstrated that IpaA disrupts focal adhesions, possibly by preventing vinculin from associating back with talin [17].

With respect to VBS-containing pathogenic proteins, it is noteworthy that sca4 in *Rickettsia* species also has two VBSs and shows a mode of vinculin activation similar to that of IpaA. Sequence alignment data show that sca4 VBSs share strongly conserved residues with IpaA VBSs, although there is no sequence conservation between the two proteins beyond the two VBSs. Thus, it is conceivable that IpaA and Sca4 share the same role in pathogen internalization, despite their differences in overall structure [56].

Vinculin activated by full-length IpaA binds to and depolymerizes F-actin [17]. In addition, a complex comprising vinculin and the IpaA carboxy-terminal domain (residues 559–633) has partial capping activity of the barbed end (plus-end) of F-actin. This finding indicates that IpaA can regulate actin polymerization by controlling the addition or removal of actin monomers [42]. However, the same IpaA construct lacks the actin depolymerization activity, implying that the actin depolymerization activity is independent of the partial capping activity. It is unclear whether the partial capping activity is directed by IpaA or vinculin. However, a recent report by Le Clainche *et al.* suggests that the barbed end capping is an intrinsic activity of vinculin, which could be induced by IpaA [57]. Actin depolymerization by the vinculin:IpaA complex is thought to be critical to form invaginations at the intimate contact sites of an otherwise repulsive surface, because of massive microspikes through uncontrolled actin polymerization induced by the activity of IpaC [17]. Partial capping activity of the vinculin:IpaA complex might provide another mechanism to control actin dynamics with the help of other actin-binding proteins [42]. More studies are needed to explain the mechanism by which IpaA modulates actin dynamics in relation to *Shigella* invasion.

## 3. IpaC-Dependent Actin Polymerization and Ruffle Formation

The Fak/Src complex controls the signaling pathway involved in cell motility, survival and cell cycle progression. Cell adhesion-dependent autophosphorylation of Tyr397 in Fak promotes Fak/Src complex formation [58]. This interaction leads to cross-activation of Src and Fak through a combination of structural changes and covalent modification of key residues in the complex. The active Fak/Src complex has multiple substrates and various cellular functions. Motility-promoting signaling depends on the activation of P130Cas and downstream Rac GTPase [59] (Figure 1).

IpaC has an amino-terminal signal sequence followed by a chaperone-binding site, two transmembrane helices and a carboxy-terminal actin polymerization domain (Figure 3A). IpaC forms a complex with IpaB in the extracellular medium, which serves as a ligand for α5β1 integrins and CD44 expressed on host cells. This interaction promotes the initial anchorage of bacteria to host cells [60]. The IpaB:IpaC complex also forms the translocation channel in the host cell membrane, which allows other effectors to be transferred into the cytosol [43].

IpaC induces actin polymerization during *Shigella* infection, independent of IpaB. An antibody directed against the carboxy terminal domain of IpaC inhibits *Shigella*-induced actin focus formation and polymerization, suggesting that the carboxy-terminal domain is responsible for such activity [19]. More recently, it has been shown that the 72 residues of the carboxy terminal in IpaC are responsible for ruffle formation and entry focus induction, which are dependent on actin polymerization [20]. The precise mechanism of action is still vague, but there is firm evidence that Src is involved in this process. Furthermore, the activity may be exerted through a direct interaction between IpaC and Src kinase. A recent report from Terry *et al.* showing that Cdc42 or Rac1 has no direct interaction with IpaC supports the possibility that Src is indeed the target of IpaC [61]. The signaling pathway directed by IpaC and Src requires further delineation.

Apart from its actin polymerization activity, IpaC also possesses an actin nucleation activity [62]. Mutation analysis by Terry *et al.* demonstrated that the 18 residues (345–363) of the carboxy terminal in IpaC are responsible for IpaC-induced actin nucleation [61]. SipC, a homolog of IpaC in *Salmonella*, has high sequence identity with IpaC at the carboxy-terminal region. A recent report by Myeni *et al.* presents evidence that SipC directly binds to and bundles F-actin through amino acid residues 221–260 and 381–409, which contribute to *Salmonella* invasion [63]. Thus, the nucleation activity region of IpaC corresponds to part of the bundling activity region in SipC. Whether IpaC also has an actin bundling activity remains to be tested.

## 4. IpgB1 and IpgB2: Bacterial Guanine Nucleotide Exchange Factors

The Rho family of GTPases is critical for controlling actin polymerization [9]. Activation of this family of proteins, including Cdc42, Rac1 and RhoA, triggers the formation of filopodia, lamellipodia and stress fibers, respectively [44]. A Rho GTPase functions as a bi-molecular switch depending on the liganded state, *i.e.*, the GTP bound is “active” and the GDP bound is “inactive”. The interchange between these two states is mediated by two groups of regulatory proteins, guanine nucleotide exchange factors (GEFs) and GTPase-activating proteins (GAPs). The GTP-Mg^2+^-binding region of the Rho GTPases consists of a phosphate-binding P-loop and flexible loops, called switch I and switch II [64]. GEFs facilitate the release of GDP for GTP to activate Rho GTPases, whereas GAPs accelerate the slow intrinsic activity of GTPases, thereby inactivating them. GEFs interact with Rho GTPases through the switch I and II regions, inducing a conformational change and subsequent release of GDP from the Rho GTPases [64].

Numerous T3SS effectors modulate actin dynamics by targeting Rho GTPases. WxxxE effectors are the largest group of such proteins, which are found in enteroinvasive pathogens [44]. WxxxE effectors are bacterial proteins of roughly 200–300 amino acid residues in length with a strictly conserved Trp-xxx-Glu sequence motif, which include IpgB1 and IpgB2 of *Shigella*, SifA and SifB of *Salmonella*, Map and EspM of pathogenic *E. coli* and EspT of *C. rodentium* [21,65,66].

A recent study by Handa *et al.* showed that IpgB1 activates Rac1 through the ELMO-Dock180 pathway, suggesting that WxxxE effectors directly mimic Rho GTPases [67]. More recent crystal structures and biochemical studies of Map and IpgB2 in complex with CDC42 and RhoA, respectively, rebutted this view and showed that WxxxE effectors directly interact with Rho GTPases to function as a GEF [68,69]. Surprisingly, the crystal structures also revealed that Map and IpgB2 are structurally and functionally similar to SopE, another bacterial GEF from *Salmonella*, which has no sequence homology with WxxxE effectors [70]. The precise mechanism of action of WxxxE effectors may be deduced from the reported structural studies of SopE, Map and IpgB2. The overall structure of these bacterial GEFs consists of 6~7 α-helices that are divided into two α-helix bundle domains. The two bundle domains orient to make a V shape, which are linked together with a stretch of amino acid residues known as the “catalytic loop”. Binding of a WxxxE effector induces a conformational change in the switch regions of the Rho GTPase due to insertion of the catalytic loop to the bound GDP-Mg^2+^ This conformational change leads to destabilization of Mg^2+^ coordination and release of GDP [68,69] (Figure 3C). Although there is no structural resemblance, the mechanism of action of WxxxE effectors functionally mimics that of the Dbl family of eukaryotic GEFs, which induce similar structural changes in GDP-Mg^2+^-bound Rho GRPases.

The GEF activity of IpgB1 and IpgB2 has been suggested to be responsible for the modulation of the host cell cytoskeleton and membrane ruffle formation during *Shigella* invasion [21,71]. In separate functional studies, the invasive capacity and the pace of invasion were reduced by 50% and 75%, respectively, by *Shigella* strains with IpgB1 deletions [22,72]. However, deletion of IpgB2 did not cause any phenotypic change in the invasive capacity [22,71]. In accordance with these results, the IpgB1/IpgB2 deletion mutant showed strong attenuation in the Sereny test and a murine pulmonary model, whereas single IpgB2 deletion mutants showed no apparent phenotypic change, suggesting dominant and unique role of IpgB1 during the early stage of *Shigella* invasion [22]. Interestingly, IpgB1 deletion alone enhanced the proinflammatory response, raising questions about the precise role of IpgB1 and IpgB2 in the pathogenesis of *Shigella* infection [22].

## 5. IpgD: Phosphoinositide Phosphatase Activity and Beyond

Phosphorylation of the inositol ring of phosphatidylinositol (PI) at the 3-, 4-, 5-hydroxyl groups can generate seven distinct phosphoinositides that play key roles in a variety of cellular functions, such as regulation of cell survival and proliferation, reorganization of the actin cytoskeleton and intracellular membrane trafficking [73]. A complex set of phosphoinositide-metabolizing enzymes, such as kinases, phosphatases and synthases, accurately regulates the level of these minute lipids. IpgD is a phosphoinositide phosphatase with the greatest enzymatic activity toward PI(4,5)P2 to produce PI(5)P [23]. However, its substrate specificity is low compared with that of mammalian phosphoinositide phosphatases, suggesting that IpgD is capable of shifting the balance of other PIs. Because the physiological levels of PI(5)P are very low compared with those of other PIs, the physiological role of this molecule has been overlooked until recently [74]. However, recent findings show that there are at least two conserved pathways dedicated to the production of PI(5)P in eukaryotic cells, suggesting that this understudied phosphoinositide may have important roles in cellular regulation and membrane trafficking [75,76]. Although the mechanism involved in this process is not clearly understood, localized production of PI(5)P at the entry site of *Shigella* appears to be critical for Akt phosphorylation followed by activation of a class IA phosphoinositide 3-kinase (PI 3-kinase) [24]. The PI 3-kinase/Akt pathway is a key regulator of cell survival [77]. IpgD-dependent activation of this pathway protects infected host cells from undergoing apoptosis to maintain efficient bacterial colonization [24].

The phenotypes of transient expression of IpgD in HeLa and NIH-3T3 cells show membrane blebbing and the disappearance of actin stress fibers with a significant decrease in membrane tension [23]. Moreover, the membrane tether force is inversely proportional to the amount of IpgD expressed in a cell. PI(4,5)P2 is the most abundant PI in eukaryotic cells, and its cellular functions include the generation of secondary messengers, exocytosis, endocytosis and reorganization of the actin cytoskeleton [73]. PI(4,5)P2 promotes actin filament assembly through direct interactions with actin-binding protein, such as N-WASP, talin and vinculin [73]. Thus, the phenotype of transient expression of IpgD is likely caused by depletion of PI(4,5)P2 rather than an effect of the newly synthesized PI(5)P. On the other hand, PI(5)P inhibits ATP release from *Shigella*-infected epithelial cells by blocking connexin hemichannels [78]. Extracellular ATP acts as an endogenous danger signal. Thus, PI(5)P produced by IpgD helps *Shigella* to avoid the early immune response by ameliorating host inflammatory responses.

*Salmonella* has an IpgD homolog, SopB (also known as SigD), which prefers PI(3,4,5)P3 and PI(3,5)P2 over PI(4,5)P2 as its substrates [29,79]. A recent report by Bakowski *et al.* proposed a new mechanism of action for SopB based on their observation of surface charge-dependent protein association with cell membranes [80]. SopB-dependent dephosphorylation of PI(4,5)P2 causes charge alteration of the membrane and inhibits recruitment of host cell proteins involved in endocytic trafficking. These events help the bacteria in *Salmonella*-containing vacuoles to avoid lysosomal degradation [31]. This study also showed that IpgD can functionally complement SopB activity. However, whether a similar strategy of membrane charge alteration is beneficial to *Shigella* invasion remains to be investigated. Another insight into IpgD functionality came from a study on SopB by Patel *et al.*, who discovered a membrane association of SopB though a putative transmembrane (TM) domain located at residues 288–309 [30]. The study also identified ubiquitin-dependent subcellular localization of SopB. Although there is no direct evidence of ubiquitination of IpgD, a sequence search showed that the TM domain is conserved between IpgD and SopB (Figure 3D). Thus, it is tempting to speculate that IpgD may also target the membrane and cause localized disturbances.

## 6. Perspectives

Here, we briefly overview *Shigella* invasins and their host cell targets (Figure 1). Although questions still remain, the biochemical and structural studies discussed here deepen our understanding of the mechanisms of how each invasin subverts its host cell target. These studies also provide insight into host cell processes involving the actin cytoskeleton. Because the functionality of invasins is associated with their redundant activities and opposing actions, a major challenge is understanding how these effector proteins synergistically collaborate to trigger efficient invasion. How does *Shigella* orchestrate the fine-tuned interplay of the effectors? Is there temporal and spatial regulation of these effectors? If so, how are the effectors controlled for productive modulation of the actin cytoskeleton? Research addressing these questions will benefit from recent advances in live-cell imaging techniques coupled with genetically-encoded fluorescent reporters and conventional Forster resonance energy transfer assays. Deeper knowledge acquired from these studies will also provide a better understanding of the invasive mechanisms of other enteroinvasive bacteria.

Among the invasins discussed here, a detailed structural study has only been performed for IpgB2. Structural studies for understanding the molecular mechanisms of other invasins through X-ray crystallography or NMR spectroscopy analyses need to be pursued. A continuous search for new virulence effector proteins that modulate the actin cytoskeleton will also help unveil the complex interplay between *Shigella* and host cells. Consequently, new insights can be gained into the development of therapies for this deadly pathogen.
